# Mr. Lowndes, on Medical Electricity

**Published:** 1807-03

**Authors:** Francis Lowndes

**Affiliations:** No. 42, St. Paul's Church Yard


### *To the Editors of the Medical and Physical Journal.*

Gentlemen,

THAT there is a fashion in Medicine, as well as in other pursuits of life, will be readily admitted by every one who has attended to the History of Physic, from the earliest ages to the present time. The love of novelty is natural to the human mind, and it enters equally into the more serious, as into the more trifling objects of our attention. Though this principle is, perhaps, wisely implanted in us, it is certainly liable to be carried too far, particularly in regard to these subjects, where perseverance and industry are essential, to carry the knowledge of them to perfection.

In perusing your useful publication, I have been often struck with this truth, on finding the highest encomium passed on a particular, too often trifling, remedy for the cure of certain diseases, while its reputation has proceeded no further than from the authority of its encomiast, and immediately afterwards it has been consigned to that oblivion from which it was only drawn by the hasty exaggeration of a sanguine enthusiast; I do not, however, mean to say, that favourers of new remedies are to be any further condemned, than withdrawing the attention from those already known, the effects of which have been proved beneficial, while they only want the united efforts and perseverance of individuals, to bring their application to that ultimate success, which might render perfect the science of medicine. This reflection is suggested to me, by contemplating the history of one remedy of a very powerful nature, the influence of which, in the cure of disease, has never been fully or properly appreciated. The power I mean is Electricity, and I shall take the liberty of introducing in the present communication, some observations on the causes which have hitherto retarded its progress, as a successful and permanent means of cure, applicable chiefly to the relief of chronic maladies.

This cause may be reduced to three heads.

1st. The too limited power of the machines in use.

2nd. Want of sufficient nicety or precision in the application, suited to the particular nature of every disease.

3dly. Deficiency in point of experience, or an unacquaintance with the diseases to which the power is specially adapted.

The introduction of Electricity into medicine, first took place as a philosophical discovery, and its application was of course consigned rather to the hands of the philosopher, than to those of the apothecary. This, for a time, could not fail to retard its progress in a medical view; but at last, it surmounted many of the difficulties, and became a very general remedy in the hands of certain practitioners, both in this country and on the continent, who devoted themselves entirely to its application. Although by this means, the objections to its frequent use became in a great measure removed, yet from the causes already stated, its success has not been equal to what might have been expected, and on these I shall now offer some remarks.

On the first, or the limited power of the instruments in common practice, I have to observe, that electricity, in order to be successful, must be conducted by a machine of such extent, that it may not be necessary to apply it in a manner violent, or forcible.

The instruments that possess this extent of power, are, indeed, very few in number, and the common ones in practice, however they may be capable of relieving some of the lesser complaints to which electricity is applied, will generally aggravate, rather than afford relief to those of a more serious and rooted nature. It was a conviction of this deficiency, soon after entering into business, that induced me to set about the construction of a machine on a more enlarged scale, than any one ever thought of in this country, the result of which has fully answered my expectation, and my practice has accordingly been attended with the most marked success in a variety of cases, which had resisted the best efforts of other practitioners with the common machines, the effect of which had even been in some of these cases, rather to increase than relieve the malady. A little attention will also shew the justice of this. The cure of diseases, it is well known, is rather affected by slow and gradual means, than by violent and hasty applications; Nature, in all her efforts to recovery, demonstrates this, and the steps of Nature should be as closely followed as possible, in the treatment of every malady; but even independant of this consideration, however important, the feelings of patients also claim attention, for whatever appears to act by violence, or in a manner deranging the ordinary train of sensation, is apt to give alarm, and to prevent the repetition of the same means, with that alacrity they would otherwise be resorted to; hence the idea of a shock, or a severe exertion of the the electric power, being an essential point to its success, as a remedy, has been a bar with too many of delicate irritable nerves to its use at all.

The second cause specified, that has retarded the progress of electricity, regards its application. The mode of application must be suited by certain contrivances to the part of the body affected, that no portion of the electric fluid may be lost; though this may be easily shewn when the machine is at hand, it cannot be so readily described to a reader, but the importance of it will sufficiently strike every one who is conversant in the practical part of the business; in local diseases, especially those of a muscular nature, it is of the first consequence. Their seat, extent, and connection with the surrounding parts, should be previously marked out, as the success of the application will depend upon it, in order that the application may be conveyed with the fullest energy.

The third circumstance enumerated above, as having retarded the progress of electricity, was deficiency in point of experience; and in support of this I may observe, that until a person is fully accustomed to the application of electricity in diseases, he will often fail in his attempts to cure; nay, the same disease, when once experienced to attack it, a practitioner will remove, though it has baffled his skill in several preceding cases, from his being unaccustomed to treat it. This particularly occurs in affections of the organs of sense, as blindness, deafness, &c.; farther, there are a number of inferior minutiæ, which are only acquired by practice, and which cannot be conveyed by information, or any of the common modes of communicating instruction on the subject. To be convinced of this truth, a person must both see and apply the power himself. Perhaps to this point may be applied the just retort of the Italian musician, to an amateur who had purchased from him at a high price, a Cremona fiddle of fine toned expression, which had failed to shew these powers in the hands of the purchaser. "I suppose, Sir, you thought to have had my fingers into the bargain." It is on this account, the necessity for electricity, forming a separate profession, will be obvious. A man accustomed to dwell on the same subject, if acquainted scientifically with its powers, cannot fail to bring it to a perfection, of which another can have no conception. He will then be able to decide on the time, frequency, and force, of the application in particular cases, with a precision and certainty of success; and even beforehand, he can offer his opinion with a confidence which another slightly skilled cannot.

To illustrate the propriety of these remarks in a still more convincing manner, as facts will ever precede opinion, however just the reasoning that may be employed to support them; I shall now in this, and perhaps some future communication, select a few appropriate cases of patients, where a successful cure was made by attention to the circumstances pointed out, after every other means directed by the first science in medical practice had failed; nor shall I draw these cases from the inferior walks of life, but from patients of elevated rank, which gave them the command of every means of relief, before coming under my care. In doing this, an experience of upwards of twenty years furnishes me with ample opportunities.

One of the first diseases I shall notice, is chronic rheumatism, from the frequency of its attack, in this country, the obstinacy of its continuance, and the constant unremitting pain which attends it.

### CASE.

The celebrated Mrs. Siddons, so long the admiration of the public for her transcendent ability and private worth, was in the course of her professional vocation, subjected to a violent attack of rheumatism in the loins, which extended on the right side from the hip to the ancle, heel, and toes: so severe was her illness, as to confine her entirely to her bed, without being able to move or rest from incessant pain. In this situation, she put herself under the care of two gentlemen, deservedly at the head of the profession, Sir Lucas Pepys and Sir James Earle, who exhausted every means, which their great skill and experience could suggest, to relieve her; but in spite of their efforts, her complaint every day gained ground, and appeared of a very rooted nature. The present attack had been preceded, about two years before, by a constant sense of coldness and torpor of the lower part of the spine and adjacent parts, the unpleasant sensations of which, had occasioned her to be constantly drawing near the fire, in order to relieve herself by presenting her back to the heat.

During the time of this slight indisposition, she was in the course of her professional duty, called on to perform the part of Desdemona, in Othello, and hence arose the violence of her malady. The stage is at all times, a situation ation of infinite peril to the performers, from the great current of air that flows over it; but, in this instance, she had to encounter an unforeseen danger; the sheets in the bedchamber scene, from the carelessness of the servants, she discovered, but unfortunately too late to admit of a remedy, to be exceedingly damp; before the conclusion of the scene she felt the ill effects; a cold chill pervaded the whole of her body, and she was, from thence, every day worse. In the course of her illness, with the above gentlemen were joined in consultation, Dr Pitcairn and Dr. Batty, the latter of whom, very properly, proposed electricity, as the remedy best suited to her complaint.

When she put herself under my care, she had been ill some months, and almost the whole of that time confined to bed, or bed-room, without the power of motion, or any relief from pain but what was afforded by large doses of opium. After the four first applications of electricity, she was enabled to move her foot and ancle, and about the tenth operation, she was so far recovered as to be able to be moved out of bed. By such successive application, the pain became gradually lessened, so that the doses of opium were daily diminished, and, at last, entirely given up. In the space of two months, her cure was completed; and since that time, she has been able, without interruption, to pursue her professional engagements as formerly.

That this case was well adapted for the powers of electricity to remove, was very justly inferred by Dr. Batty from the preceding history of torpor and diminished sensibility, which affected the whole of the limb and loins. The nervous influence was here chiefly in fault; its diminished energy, whatever the peculiar morbid state on which this depends, was only to be removed by a power which could assimilate itself with the nervous influence, and act more forcibly and independantly upon it than the other parts of the animal structure. I am, &c.

*No. 42, St. Paul's Church Yard,* FRANCIS LOWNDES.

*February* 16, 1807.

